# Effects of ZnO Nanoparticle on the Gas Separation Performance of Polyurethane Mixed Matrix Membrane

**DOI:** 10.3390/membranes7030043

**Published:** 2017-08-11

**Authors:** Banafsheh Soltani, Morteza Asghari

**Affiliations:** 1 Separation Processes Research Group (SPRG), Department of Chemical Engineering, University of Kashan, Kashan 8731753153, Iran; b1soltani@yahoo.com; 2 Energy Research Institute, University of Kashan, Ghotb-e-Ravandi Ave., Kashan 8731753153, Iran

**Keywords:** polyurethane (PU), ZnO nanoparticle, mixed matrix membrane, gas separation

## Abstract

Polyurethane (PU)-ZnO mixed matrix membranes (MMM) were fabricated and characterized for gas separation. A thermogravimetric analysis (TGA), a scanning electron microscope (SEM) test and an atomic-force microscopy (AFM) revealed that the physical properties and thermal stability of the membranes were improved through filler loading. Hydrogen Bonding Index, obtained from the Fourier transform infrared spectroscopy (FTIR), demonstrate that the degree of phase separation in PU-ZnO 0.5 wt % MMM was more than the neat PU, while in PU-ZnO 1.0 wt % MMM, the phase mixing had increased. Compared to the neat membrane, the CO_2_ permeability of the MMMs increased by 31% for PU-ZnO 0.5 wt % MMM and decreased by 34% for 1.0 wt % ZnO MMM. The CO_2_/CH_4_ and CO_2_/N_2_ selectivities of PU-ZnO 0.5 wt % were 18.75 and 64.75, respectively.

## 1. Introduction

From an environmental standpoint, CO_2_ is one of the most significant greenhouse gases and its elimination is necessary, as it is responsible for global climate change [1,2].

The most common approach for CO_2_ elimination is solvent absorption. High-fixed investment, operating cost, phase distribution, and the mass transfer surface limit are posing impediments to this process [2,3].

Recently, one of the techniques considered to be a suitable alternative for CO_2_ separation is the execution of the membrane process without the necessity of phase change. This will provide the separation with less energy consumption [4]. Nowadays, membrane processes are one of the most pioneering technologies for gas separation, specifically in the chemical industry, due to its lower processing and initial cost, lower energy consumption, and less space requirements [5,6,7,8]. The use of polymeric membranes in capturing CO_2_ has drawn the attention of many scientists [9,10,11]. This is due to their high permeability and/or selectivity. Presently, membranologists are trying to develop novel polymer structures to improve the membrane separation properties. However, permeability and selectivity behaviours of the usual polymeric membranes indicate trade-off trends [10]. These years, studies on the structure of the membrane to increase their efficiency continue. Mixed matrix membranes (MMMs) are polymeric membranes filled with (nano)particles uniformly distributed in the polymer matrix to increase the membrane performance. The possibility of using organic or inorganic composite materials has been examined by researchers to improve the properties of the MMMs [12,13,14,15].

Polyurethane (PU) is a polymeric material with a wide range of applications, including adhesive, fibre, foam, thermoplastic elastomer, and coating. Its specific structural properties are affected by its two-phase structure—the hard urethane and the soft polyester or polyether segments. PU hydrogen bonding competes between the two PU segments. The crystallisability of these segments change the structural features of the PU. Nanoparticles’ interaction with these segments change the balance of the hydrogen bonding between the hard (HS) and soft segments (SS). Then, the mixing or separation phase occurs [16,17,18]. The results of the incorporation of phase mixing or separation affect the physical and chemical properties of the PU.

These special properties of the PU produce good permeation properties for the gas separation system, as well as the good physical, mechanical and thermal properties, fatigue life, and abrasion resistance. Thus, the PU membrane has allocated a lot of the gas separation membrane studies in recent years [16,17,18,19,20,21,22,23,24]. Many organic and inorganic materials have been used as fillers to incorporate into the PU membranes [25].

Bistricic et al. studied the PU-nanosilica MMM. They reported that the hydrogen bond formation between the nanosilica and the carbonyl groups in the soft segments of PU improved the rheological, thermal, mechanical and adhesive properties of the PU-nanosilica MMM [16].

In another work, Khosravi et al. examined the role of the silica nanoparticles in the polyether and polyester of the PU MMMs for gas permeation [26]. They reported that the polyether urethane-silica membranes had higher propane permeability and propane/methane selectivity. The propane permeability and propane/methane selectivity of the polyester urethane decreased due to nanosilica loading [26]. Some of the PU MMMs with different nanoparticles for gas separation are shown in Table 1.

Formerly, Iron oxide and ZnO catalysts have been used to sweeten sour gas. The use of metal oxide nanoparticles can greatly increase the absorption processes due to its physical and chemical properties [31]. ZnO could undergo hydrogen bonding with PU. The amines of HS and carbonyls of both HS and SS could be interacted with ZnO and its O–H groups to form hydrogen bonds and affect the PU structure. Therefore, in this study, ZnO nanoparticles were applied to improve the separation performance of PU membranes.

The use of nonporous ZnO nanoparticles as fillers increased the anti-bacterial, mechanical, physical and optical properties of the polymeric composite [25]. Kim et al. added the ZnO nanoparticles to the polyurethane acrylate nanocomposite film to enhance its optical, physical and mechanical properties, and water transport behaviours. However, the thermal stability of the nanocomposite film was observed to be weak [32].

Thus, the gas permeation properties of a tiny amount of ZnO on the PU composite membrane were investigated. As it is observed in Table 1, all the studies on PU MMM have used 5–25% of nanoparticles. In this present study, the effects of the incorporation of a tiny amount of ZnO nanoparticles into the PU MMMs and their gas separation properties were investigated for the first time.

The gas separation properties of PU-ZnO MMMs and the effect of ZnO as a metal oxide on the soft and hard segments of PU were also investigated for the first time. Different amounts of ZnO nanoparticles were added to the PU matrix to study the membranes’ permeability for CO_2_, CH_4_, and N_2_. Moreover, the membrane samples were characterised using precise analysing methods.

## 2. Experimental

### 2.1. Materials

Polyester urethane (PU) with the commercial code (Apilon52-A6505) was supplied by API SpA, Neu-Isenburg, Germany. *N*,*N*-dimethylformamide (DMF) was purchased from Merck, Darmstadt, Germany. ZnO nanoparticles (purity > 99%; particle size range 20–30 nm) were supplied by Navarrean Nanoproducts Technology (TECNAN, Navarra, Spain). All of the chemicals and materials were used as received.

### 2.2. Membrane Preparation

The PU and PU-ZnO MMM samples were prepared using solving, casting and solvent evaporating methods.

A sufficient amount of PU was added to the solvent (DMF) and the obtained dope solution (10 wt %) was stirred to get a homogeneous PU solution. The dope solution was then cast on glass plates and the solvents were allowed to evaporate.

In order to allow better evaporation of the solvent from the membranes, the casted films were removed from the glass plates and dried slowly in a vacuum oven. The PU-ZnO membranes were prepared using the same method. The ZnO particles were well dispersed in a mixture of half DMF and 1 wt % of the PU. The target mixture was mixed for about 12 h. Another PU-DMF solution was made and the dope solution, containing ZnO, was added into it. Then, the final mixture was mixed well, sonicated for 20 min, and cast as PU dope.

### 2.3. Characterization of the Membranes

The IR spectroscopy (Magna IR Spectrometer 550, GMI, Ramsey, MN, USA) was used for monitoring the interaction between the PU and ZnO nanoparticles in the MMMs and confirm the final chemical structures of the membrane samples. The scanning frequency range is 4000–400 cm^−1^.

The membranes’ morphology and the distribution of ZnO nanoparticles in the MMMs’ structures were investigated using the scanning electron microscopy (SEM) (TESCAN, Brno, Czech Republic). The MMM samples were well-fractured in liquid nitrogen and coated with gold before the cross-sectional scanning.

The surface topography of the control sample (neat PU membrane) and the PU-ZnO membranes was studied using the atomic force microscopy (AFM) (SOLVER, NT-MDT, Moscow, Russia) via non-contact mode.

The thermal degradation analysis of the nanocomposite membranes was done through the thermal gravimeter/differential thermal analyser (TG/DTA) (PYRIS DIAMOND, Perkin Elmer, Waltham, MA, USA). The results were obtained from 25 °C to 600 °C at a heating rate of 10 °C/min.

### 2.4. Permeation Measurements

The gas permeability performance of the membranes was measured using a constant pressure system. The membrane permeability (*P*) can be calculated using the Equation (1):

P = Q* × t/(A × (p − p°)) (1)

In this equation, the feed pressure (*p*) varies from 4 to 14 atm, and the permeate pressure (*p°*) is atmospheric. Equation (1) is normalised with the partial pressure difference (*p − p°*). The flux (*Q*) is measured using a bubble flow meter in a steady state flow. The temperature was maintained at 30 °C and *t* is the membrane thickness, which is measured using the SEM images about 35–38 µm. The membrane effective module area in this work was 27.72 cm^2^. Moreover, the membrane permeability is expressed in barrers [1,4,18,19,20,29,33].

## 3. Results and Discussion

### 3.1. IR Analysis

**Figure 1:**
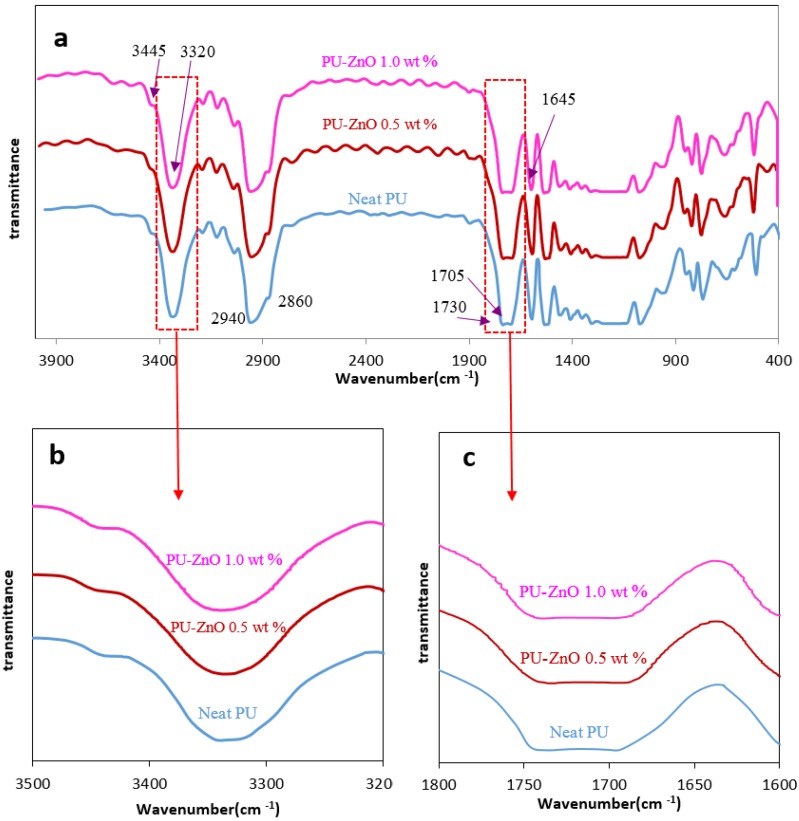
IR analysis (**a**) IR analysis of PU-ZnO MMMs; (**b**) NH segment; (**c**) C=O segment.

The chemical properties of the neat PU membrane and the ZnO-filled MMMs are characterised by conducting the IR analysis. The IR results are shown in Figure 1. The PU is naturally hydrogen-bonded and its general structure (–CO–O–R–O–CO–NH–R–) contains both the hard segments (–R–O–CO–NH–R–) and the soft segments (–R–CO–O–R–). The HS of urethane is hydrogen-bonding. The carbonyls of either the HS (i.e., urethane) or the SS (i.e., ester) can form these hydrogen-bonding structures. The phase separation degree determines these two places of the hydrogen bonds in the urethane’s structure. The changes of band intensities in N=H (3300–3500 cm^−1^) and C=O (1600–1750 cm^−1^) regions are usually studied using the IR spectra [16]. The hydrogen bonding measurement of the urethanes in the HS and the carbonyl in both the HS and the SS can show the presence of the nanoparticles in the PU matrix as well as the degree of phase mixing or separation. The major IR peaks of the PUs are shown in Table 2.

The hydrogen bonding quantity of the N–H groups can be investigated using the spectrum of the N–H stretching region. The intensive peak at 3330 cm^−1^ for the PU MMMs shows the intermolecular interactions occurring in the HS between the carbonyl and the amine groups (N–H⋯O=C_urethane_). The shoulder on 3440 cm^−1^ shows the vibrations of N–H in the hard segments that can form the hydrogen bond with the carbonyl of the SS(N–H⋯O=C_ester_) [20,26]. As it is shown in Figure 1a, the intensity of the N–H bonds in the PU-ZnO MMM, containing 0.5 wt % nanoparticles, are lower than the neat PU. This indicates that the N–H⋯O=C_ester_ stretching bond had decreased and some phase separation occurred in the PU membranes with nanocomposite structure. However, according to the measured peaks of the PU-ZnO 1.0 wt % MMM sample, the N–H⋯O=C_ester_ bond intensity is higher than the neat PU. As revealed in IR results, ZnO sits on the SS of PU and the O–H of ZnO tends to absorb the N–H of the HS to form hydrogen bonds. Therefore, by absorbing N–H on the SS, formation of N–H⋯O=C_ester_ bond becomes most possible, so the corresponding intensity increases.

Carbonyl formed different types of hydrogen bonding in the PU and the carbonyl peaks were divided into three regions. The non-hydrogen bonding of the free carbonyls is observed near 1730 cm^−1^, and the carbonyl with poorly hydrogen bonding are observed at 1710 cm^−1^, and the hydrogen-bonded carbonyl’s peak of the HS can be observed at 1690 cm^−1^ [12]. Based on the examination of the carbonyl stretching band for the PU-ZnO MMM in Figure 1b, it appears that the intensity of the free carbonyl band slightly decreases in the PU-ZnO 0.5 wt % MMM, compared to the neat PU membrane. Moreover, the position of carbonyl hydrogen band shifts a little to a lower wave number in the PU-ZnO 0.5 wt % MMM. This can detect more hydrogen bonding between the carbonyl groups and the urethane N–H groups in the HS and a partial phase separation in the PU-ZnO 0.5 wt % MMM. This may be related to the distribution of the ZnO nanoparticles in the PU soft segment [30]. In the PU-ZnO 1.0 wt % MMM, fewer hydrogen bonds were observed in the N–H and the carbonyl groups in the HS. The distribution of ZnO particles in the SS and their interaction with the carbonyl groups in this segment contributed to attracting the N–H groups to the carbonyl in the SS for higher nanoparticle concentration (1.0 wt % ZnO). This indicates the phase mixing in the proposed MMM. The amount of Hydrogen Bonding Index (HBI) and its variations in the PUs are defined as follows:

HBI = A_C=O bonded_/A_C=O free_ (2)

Here, A_C=O bonded_ and A_C=O free_ are the bonded and free carbonyls absorbance, respectively. The variation of the HBI in the MMMs was investigated with the nanoparticle contents [34]. The HBI results in Table 3 indicated that the HBI increases from 1.089 for the neat PU to 1.377 for the PU-ZnO 0.5 wt % MMM, while it decreases to 0.808 for the PU-ZnO 1.0% wt MMM. Thus, the degree of phase separation for the PU-ZnO 0.5 wt % MMM is higher than that of the neat PU. However, for the PU-ZnO 1.0 wt % MMM, a higher HBI decline was detected in phase mixing compared to that for the neat PU.

Stretching of the O–H band in pure ZnO was observed to be 3500 cm^−1^. This peak is not detectable in the MMM spectra; however, the peaks observed around 1600 and 1380 cm^−1^ occurred due to the presence of zinc carboxylate. These two regions were for its asymmetrical and symmetrical stretching, respectively [25].

Due to these observations of the MMMs IR spectra, it could concluded that the ZnO nanoparticles are more distributed in the SS, and the O–H groups of the ZnO nanoparticles interact with the ester and carbonyl groups of the SS. On the basis of the IR results, it is clear that a phase separation can induce the ZnO incorporation into the PU-ZnO 0.5 wt % MMM, while the phase mixing occurs in the HS and the SS for the PU-ZnO 1.0 wt % MMM. Therefore, it can be predicted that the permeability increases for the PU-ZnO 0.5 wt % MMM and decreases for the PU-ZnO 1.0 wt % MMM.

### 3.2. Morphological and Topographical Features of the ZnO Incorporated PU Membranes

#### 3.2.1. Scanning Electron Microscopy (SEM)

**Figure 2:**
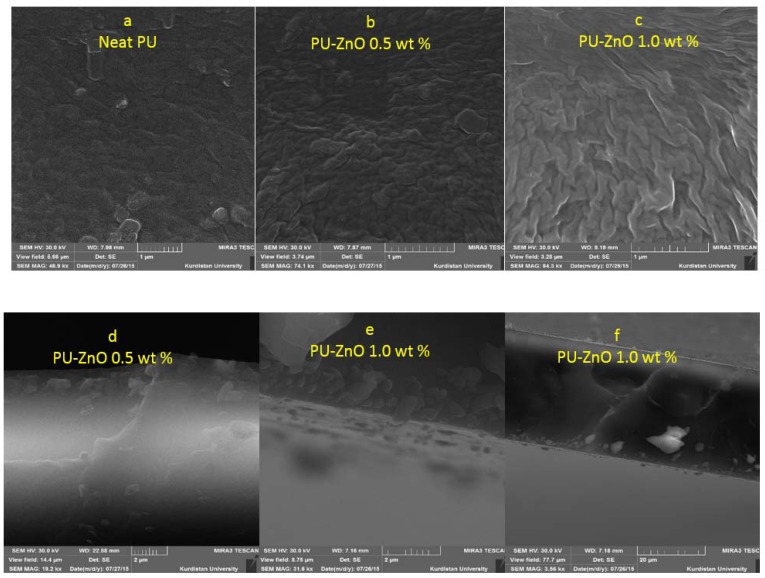
SEM surface images of MMMs (**a**) neat PU; (**b**) PU-ZnO 0.5 wt %; (**c**) PU-ZnO 1.0 wt % and cross section; (**d**) PU-ZnO 0.5 wt %; (**e**) PU-ZnO 1.0 wt %; (**f**) PU-ZnO 1.0 wt %.

The surface morphology of the PU MMMs can affect the gas separation performance. The SEM images of the ZnO-PU nanocomposite membranes are used to observe the distribution and compatibility of nanoparticles in the polymer matrix. Figure 2 shows the SEM images of the top surface and the cross-section of the PU-ZnO MMM. As observed, the ZnO nanoparticles uniformly dispersed in the polymer matrix.

The cross view of the SEM images for the PU-ZnO membranes, containing 0.5 wt % and 1.0 wt % MMM, are shown in Figure 2a,b. These images illustrate that the ZnO nanoparticles were well dispersed in the PU-ZnO MMM matrix. Moreover, the MMMs were dense due to the absence of pinholes and connected the pores and/or cracks.

#### 3.2.2. Atomic Force Microscopy (AFM)

**Figure 3:**
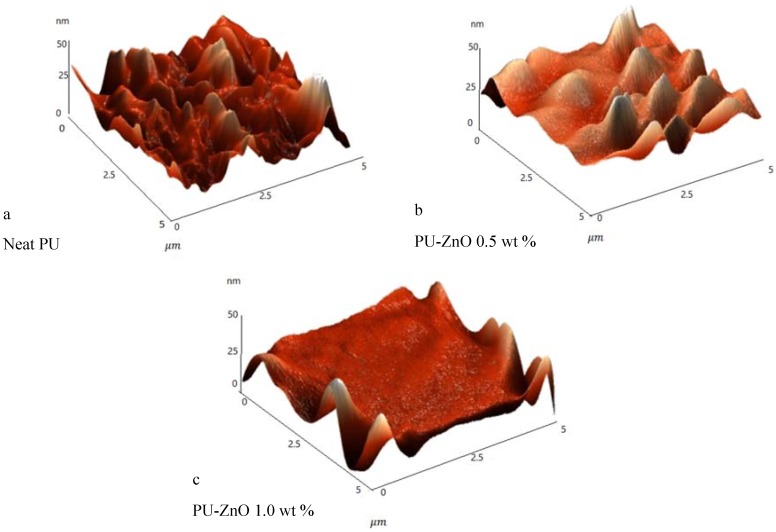
AFM images of the MMMs (**a**) neat PU; (**b**) PU-ZnO 0.5 wt %; (**c**) PU-ZnO 1.0 wt %.

A surface topography of the PU-ZnO MMM was executed using the AFM analysis. The AFM images of the PU and the PU-ZnO MMM are shown in Figure 3. As observed in these images, the presence of ZnO particles led to a smoother surface, while the neat PU membrane had a rougher surface than the PU-ZnO MMM.

According to the AFM results, it can be concluded that by increasing the ZnO concentration from 0.5 wt % to 1.0 wt % of the polymer, the surface roughness for the neat PU decreased from 12.29 nm to 3.38 nm and 1.15 nm for the PU-ZnO membrane samples, containing 0.5% and 1% nanoparticles, respectively.

The ZnO particle size is in the range of 20–30 nm, almost the same as the distance between the polymeric branches [23,24]. Consequently, the nanoparticles might easily be placed within the polymer matrix without creating more space and decreasing the surface roughness.

In addition, due to the AFM images, good adhesion in nanoparticles and polymers can be observed.

### 3.3. Thermal Stability of ZnO-Incorporated Membranes

**Figure 4:**
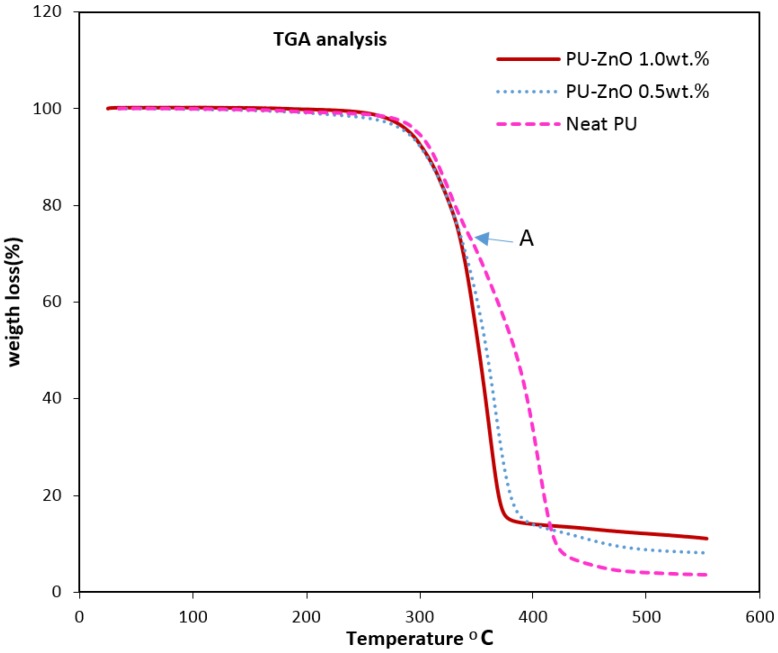
TGA analysis of PU and PU-ZnO MMMs.

Results of the TGA of the PU-ZnO MMM are shown in Figure 4. Weight loss (WL) in all membrane samples started around 270 °C and finished at 430 °C. It is worth quoting that, at 430 °C, only 3.6 wt % of the pure PU remained. At this temperature, the residual weight percentages 8.1% and 11.1% for the PU-ZnO incorporated with 0.5 wt % and 1.0 wt % MMMs, respectively.

This result is in agreement with the theoretical ZnO weight percentage of the nanocomposite membrane. Moreover, the results indicated that the ZnO-incorporated membrane samples have higher WL rates than that of the PU. The higher thermal conductivity of the ZnO particles causes the proposed higher WL rate. The TGA also indicates that no residual solvent remained in the membrane matrix.

Two different reduction slopes are observed for the neat PU in thermal degradation. The first one is related to the degradation of the HS, while the second one is related to the thermal decomposition of polyester. As shown in Figure 4, the first level of neat PU decomposition starts at 275 °C, and the second level at 345 °C (point A). As observed, this difference disappears for the ZnO-filled PU membranes; hence, the decomposition rate of the SS becomes similar to that of the HS. It could then be concluded (see IR results) that the ZnO-nanofiller would be incorporated in the SS of the polymeric membrane.

### 3.4. Gas Permeation Properties

**Figure 5:**
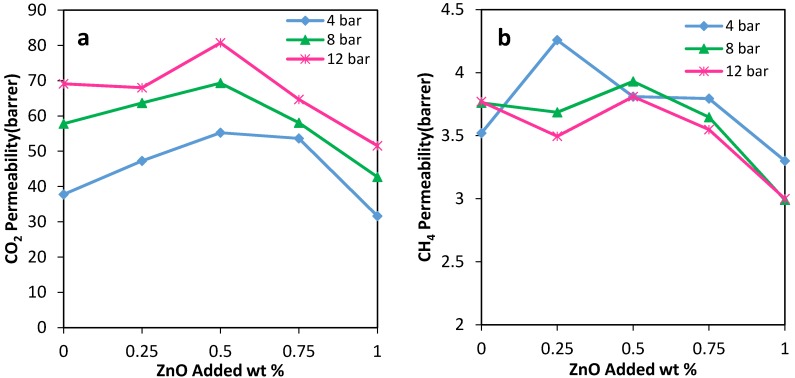

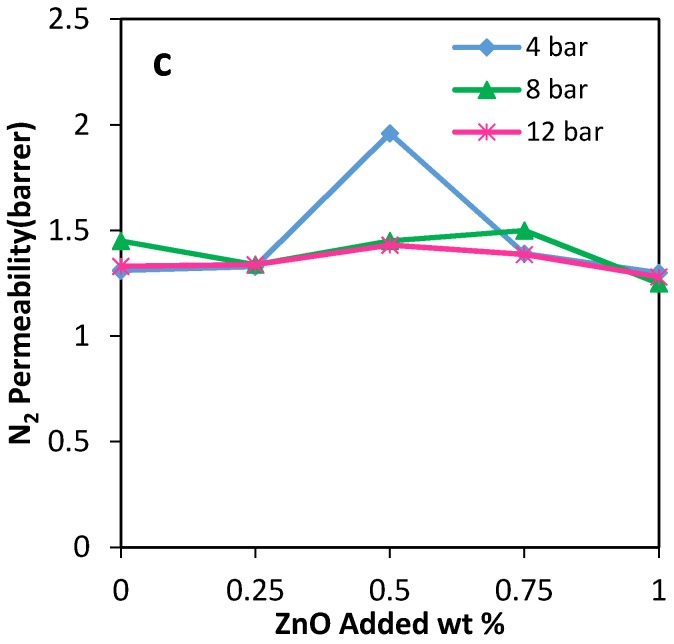
(**a**) CO_2_; (**b**) CH_4_ and (**c**) N_2_ permeabilities through the MMMs vs. ZnO nanoparticles loading in PU.

**Figure 6:**
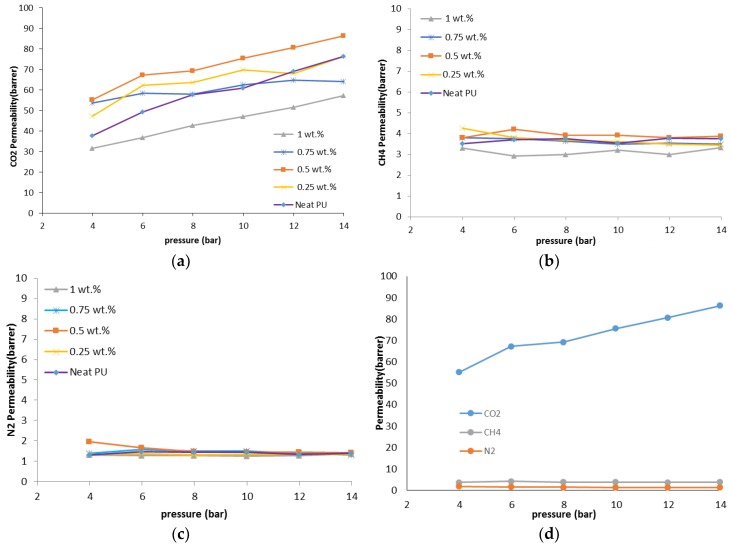
(**a**) CO_2_; (**b**) CH_4_ and (**c**) N_2_ permeabilities through the MMMs vs. pressure; (**d**) pure gas permeabilities of PU-ZnO 0.5 wt %.

The gas permeability of the MMMs is measured, respectively, for N_2_, CH_4,_ and CO_2_ as pure gases at six different operating pressures of 4–14 bar and the constant temperature of 30 °C. The CO_2_, CH_4,_ and N_2_ permeabilities of the PU-ZnO MMM are shown in Figure 5 and Figure 6 and Table 4. As could be observed in Figure 5, the CO_2_ permeability is significantly higher than that of CH_4_ and N_2_, using both the neat membrane and the MMMs. The higher CO_2_ permeability, compared to CH_4_ and N_2_, occurs due to its polarizability [35], smaller kinetic diameter, and greater condensability (see Table 5) that leads to a stronger interaction with the polar groups of the PU matrix [30,36,37,38].

**Figure 7:**
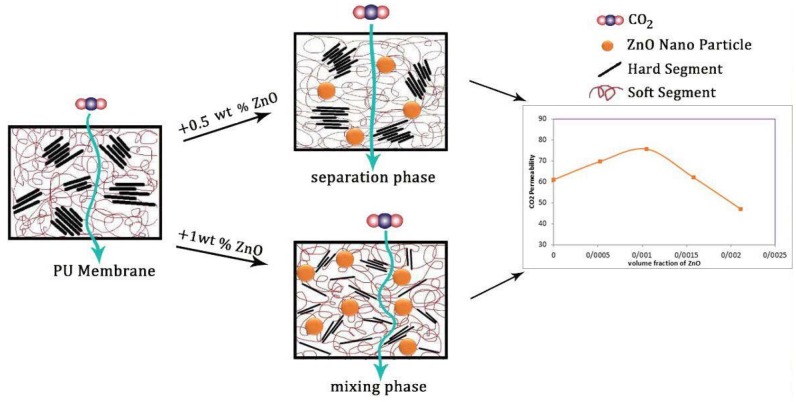
Effect of ZnO nanoparticle on the membrane permeability.

As could be observed in Figure 5a, the CO_2_ permeability of the PU-ZnO 0.25 and 0.5 wt % MMM is approximately 12% and 30%, respectively, higher than that of the neat PU membrane. However, it decreases about 34% for the membrane, incorporated with a higher amount of ZnO (1.0 wt %). Moreover, the IR and AFM results confirm these behaviours. As mentioned earlier for the IR section, the phase separation in the soft segments of the PU-ZnO 0.5 wt % MMM led to the higher gas permeation compared to the neat membrane (Figure 7). The lower permeability of the PU-ZnO 1.0 wt % MMM can be explained using the IR results. Due to the presence of 1.0 wt % ZnO, both the phase mixing in the PU segments and free volume reduction of the PU SS can cause the gas molecule diffusion pathway to be more torsional [39] (Figure 7). On the other hand, the smoother surface of the PU-ZnO 1.0 wt % MMM, as mentioned earlier in the AFM section, led to the decrease in the surface interaction of the membrane, and, consequently, lesser gas permeability. This can be explained by observing the similar trends of N_2_ and CH_4_ permeation through the PU-ZnO 1.0 wt % MMM.

**Figure 8:**
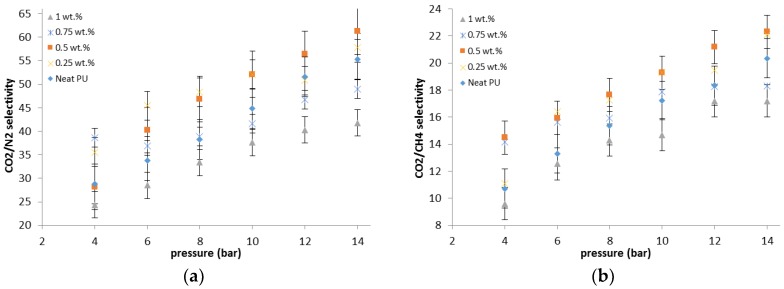
(**a**) CO_2_/N_2_ and (**b**) CO_2_/CH_4_ selectivities of PU-ZnO MMMs.

As could be observed in Figure 8, compared to the neat membrane, the CO_2_/N_2_ selectivity of the 0.25 wt % and 0.5 wt % MMMs are around 18% higher and the PU-ZnO 1.0 wt % MMMs are about 20% lower than the selectivity of the neat PU. As mentioned earlier, the ZnO nanoparticles interact with the polar groups of the SS of PU during the phase separation and formation in the PU-ZnO ≤ 0.5 wt % MMM. As can be seen in Figure 8, the CO_2_/N_2_ and CO_2_/CH_4_ selectivity of the PU-ZnO MMM increases with operating pressure. This is due to the higher condensability of CO_2_ compared to N_2_ and CH_4_. Moreover, the selectivity of the PU-ZnO 0.75 wt % and 1.0 wt % MMM is less than that of the PU-ZnO 0.5 wt % MMM. This is due to the PU phase mixing and the rigid structure of the PU-ZnO ≥ 0.5 wt % MMM. The PU-ZnO 0.5 wt % MMM shows the best CO_2_ permeability of 91.33 barrer and the CO_2_/CH_4_ and CO_2_/N_2_ selectivity values of 19.76 and 61.75, respectively.

#### 3.4.1. Effects of Nanoparticles on Separation Properties of the Membrane

The gas transport properties of organic-inorganic MMMs mainly depend on the membrane structure. The membrane structure largely depends on the surface contact between the nanoparticles and the polymer [40]. In the MMMs, the nanoparticles usually affect the membranes’ permeability in two major ways. One of them is disrupting the polymer chains, which increases the gas permeability. The other one is creating obstacles in the path of gas penetration that increase the length of the diffusion path and reduce gas permeability [41,42,43].

As mentioned earlier, the PU has two segments—soft and hard. It is worth quoting that only the soft segment affects the gas permeation [17,22]. From an entropy point-of-view, the metal oxide nanoparticles tend to be distributed in the HS. However, the IR and TGA results indicate that the nanoparticles were distributed in the SS of the PU polymer. In the proposed membranes, the amounts of ZnO nanoparticles in the polymer are 0.25, 0.50, 0.75, and 1.0 wt %, based on the polymer basis. The applied nanoparticles have a very small size with an average diameter in the range of 20–30 nm, which is almost the same as the distance between the polymer branches. Consequently, the nanoparticles are easily placed within the polymer matrix without creating more space. This proposed criterion of filling the pores of the polymer substrate by nanoparticles can also be confirmed by the AFM results.

According to the IR analysis, the mixing phase of the hard and soft segments occurs for the 1.0 wt % nanoparticle, added into the PU-ZnO membrane. This can be attributed to the dipole interactions of the ZnO nanoparticles with the functional groups of the SS. On the other hand, the interaction of nanoparticles with the PU can be described as follows.

When 0.5 wt % of the ZnO nanoparticle is added to the polymer (i.e., PU), the ZnO nanoparticle can affect the ester section, and, therefore, some links are established among the ZnO nanoparticles and the carbonyl groups. This leads to the phase separation of the SS and the HS. In fact, the nanoparticles occupy carbonyl sections and prevent the conjunction of these segments with the hard segments. Increasing the ZnO concentration up to 1.0% wt and increasing the created dipole moment in the SS provides the stretching for the absorption of amine groups in the HS to the SS of the PU. Therefore, the nanoparticles contribute to the formation of hydrogen bonds between the SS and the HS. Thus, it can be concluded that the addition of ZnO nanoparticles up to 0.5 wt % increase the permeability, while adding ZnO more than 0.5 wt % of the same decreases gas permeability.

By using the PU-ZnO 0.25 wt % and 0.5 wt % MMM, compared to the neat PU membrane, the CO_2_/N_2_ selectivity increases up to 18% (Figure 8), while, for the PU-ZnO 1.0 wt % MMM, it decreases about 20%. The CO_2_/CH_4_ selectivity is also increased up to 8% for the PU-ZnO 0.25 and 0.5 wt % membrane, while it is reduced by about 15% for the PU-ZnO 1.0 wt % MMM. As mentioned earlier, the gas permeability of the PU-ZnO 1.0 wt % MMM is lower than that of the neat PU.

The gas permeation results showed that both the permeability and selectivity were increased for the PU-ZnO 0.25 wt % and 0.5 wt %, and decreased for the PU-ZnO 1.0 wt % MMM. This can be explained as follows: (*i*) ZnO affinity to CO_2_ molecules, (*ii*) ZnO effect on the SS of the PU, and (*iii*) the effect of ZnO nanoparticles on the surface roughness of the MMMs. For the PU-ZnO 0.25 wt % and 0.5 wt % MMM, the effect of ZnO on the polymer SS (dipole-dipole interactions and IR results) caused the CO_2_ molecules to be absorbed more than the N_2_ and CH_4_ molecules. Therefore, both permeability and selectivity increased.

#### 3.4.2. The Effect of Pressure on the Membranes

Figure 6 shows the effect of operating pressure on the permeability for the PU-ZnO-MMM. As could be observed, N_2_ permeability for the proposed PU-ZnO membranes decreases with the increase in the operating pressure. For all of the investigated membranes, CH_4_ permeability decreases slowly with the increasing operating pressure from 4 to 14 bar. However, CO_2_ permeability increases, almost linearly, with the increasing pressure in the same range.

These partial declines of the permeability with the operating pressure for N_2_ and CH_4_ gases demonstrates that there is no macroscopic porosity in the membrane structure. On the other hand, there is no increase in the permeability for the proposed gases (N_2_ and CH_4_), which can confirm the dense structure of the fabricated PU-ZnO MMM. Moreover, higher CO_2_ permeability at higher pressures can refer to its higher solubility, which is due to more absorption of CO_2_ molecules in the polymeric network, as well as high compressibility of this gas in the polymer membrane [44].

Moreover, by increasing the operating pressure, CO_2_ fills the space between the chains of the polymer network. This increases the gap between these bonds, which consequently increases the mobility of the polymeric chain [45], causes the plasticity of the membranes, and also increases the membrane permeability [46,47,48]. All of these facts can be translated to the higher permeability of CO_2_ with the operating pressure.

On the other hand, a decline in the permeability of N_2_ and CH_4_ gases can be explained as follows: an increase in the operating pressure compresses the membrane; hence, the free volume decreases. As the membrane is not porous, higher pressure can decrease the gas permeability. The CH_4_ molecules have a higher compressibility compared to the N_2_ molecules. Therefore, more CH_4_ molecules can be absorbed into the polymer chains than N_2_ molecules. This can be investigated as the main reason for the decline in nitrogen permeability.

In addition, the permeability equation has two important factors: pressure in the denominator and flux in the numerator. If pressure grows, both of them grow. The increase or decrease of the permeability depend on the contest of growth between these two factors. As it is mentioned earlier, the CO_2_ molecules affect the polymer structure’s plasticity and increase the flux, but the CH_4_ or N_2_ molecules cannot do this; hence, their permeability decreases with the increasing pressure.

#### 3.4.3. Comparing the Performance of Synthesized MMMs and Other PU MMMs

**Figure 9:**
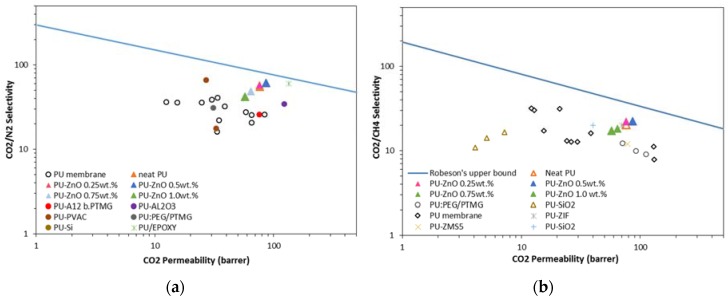
Comparison of CO_2_/N_2_ (**a**) and CO_2_/CH_4_ (**b**) separation performance of PU-ZnO MMMs in Robeson’s upper bound [19,20,33,38,42,50,51,52,53,54].

The obtained results for gas separation performance (e.g., permeability and selectivity) of the proposed PU-ZnO MMM are compared with the literature in Robeson’s upper bound limits [49]. The results are shown in Figure 9a,b. As observed, the CO_2_/N_2_ separation result for the PU-ZnO MMM approaches the Robeson line. Moreover, the data for PU-ZnO 0.5 wt % MMM is closer to the Robeson line, while the situation for the PU-ZnO 1.0 wt % MMM is farther. Although the permeability and selectivity data of the proposed MMMs do not cross the Robeson boundary, it could be observed that the incorporation of the ZnO nanoparticles into the polymeric matrix improves their performance.

Comparing the results for the CO_2_/N_2_ separation, that are shown in Figure 9a, revealed that the MMMs of this work had a higher permeability with good selectivity features compared to the PU membranes, incorporated with other nanoparticles in the literature. Figure 9b shows the CO_2_/CH_4_ separation in comparison with the Robeson line. As observed, the synthesized PU-ZnO MMM in this work have higher selectivity and permeability than that of the PU membranes, incorporated with other nanoparticles such as SiO_2_ [20], Al_2_O_3_ [19], ZIF [18], epoxy [17], and ZSM-5 [18]. All of these membranes have the same separation performance compared to the PU-ZnO MMM in this study.

## 4. Conclusions

The effects of a tiny amount of ZnO nanoparticles on the morphological and gas separation performance of the PU-based MMMs were investigated. The IR results of the neat and MMMs indicated that the incorporation of ZnO up to 0.5 wt % in the PU-ZnO MMM led to a phase separation in the membrane, while in the PU-ZnO 1.0 wt % MMM, it led to a phase mixing. The SEM images proved that the ZnO nanoparticles were dispersed well within the polymeric matrix. On the other hand, the AFM observation of the neat PU showed a rougher surface than that of the PU-ZnO MMM. The TGA indicated that the incorporation of ZnO to the PU had no thermal transience in the PU-ZnO MMM. The TGA results also demonstrated that the ZnO nanoparticles dispersed in the SS of the PU.

The gas permeation results showed that the permeability of N_2_, CO_2,_ and CH_4_ through the PU-ZnO 0.5 wt % MMM was higher than that of the neat PU membrane. The CO_2_ permeability of PU-ZnO 0.5 wt % MMM was approximately 30% higher than that of the neat PU membrane, while it decreased by about 34% for the membrane containing 1% of ZnO nanoparticles.

## Figures and Tables

**Table 1:** PU MMMs with different nanoparticle for gas separation.

Type of PU	Application	Nano Particle	Nano Particle Content (wt %)	Refs
Polyurethane	CH_4_, C_2_H_6_	Silica	0, 2.6, 6, 11.5	[8]
Polyurethane	CH_4_, C_2_H_6_	zeolite 4A	0, 10, 20	[27]
Polyester urethane	CO_2_, CH_4_	silica-Un	5, 10, 15	[20]
Polyester urethane	CO_2_, CH_4_	silica-OS1	5, 10, 15	[20]
Polyester urethane	CO_2_, CH_4_	silica-PDMS	5, 10, 15	[20]
Polyurethane	CO_2_, CH_4_, O_2_, N_2_	Alumina	0, 10, 20, 30	[19]
Polydimethylsiloxane (PDMS)–PU	CO_2_, O_2_, N_2_	POSS	5, 10, 15, 20, 25	[28]
Polyurethane	H_2_, O_2_, N_2_	TiO_2_	25	[29]
Polycaprolactone based polyurethane	CO_2_, CH_4_, O_2_, N_2_	silica	0, 2.5, 5, 10, 20, 30	[30]
Polyurethane	He, CH_4_, O_2_, N_2_	clay	10, 20, 30	[21]

**Table 2:** Major infrared bands of PUs.

Wavenumber (cm^−1^)	Assignment
3445	N–H stretching (free)
3320–3305	N–H stretching (hydrogen bonded)
2940	Asymmetric C–H stretching
2860	Symmetric C–H stretching
1730	C=O stretching (free)
1710–1705	C=O stretching (hydrogen bonded—soft segment)
1645–1635	C=O stretching (hydrogen bonded—hard segment)

**Table 3:** Hydrogen bonding index (HBI) amounts of PU MMMs.

Membrane	Neat PU	PU-ZnO 0.5 wt % MMM	PU-ZnO 1.0 wt % MMM
HBI	1.089	1.377	0.808

**Table 4:** Pure gas permeabilities of the MMMs at different pressures.

Sample	Permeability (Barrer)									
	Thickness t (µm)	4 Bar			8 Bar			12 Bar		
		P_N2_	P_CH4_	P_CO2_	P_N2_	P_CH4_	P_CO2_	P_N2_	P_CH4_	P_CO2_
Neat PU	35 ± 2	1.31 ± 0.19	3.52 ± 0.13	37.73 ± 0.10	1.45 ± 0.17	3.76 ± 0.11	57.79 ± 0.08	1.33 ± 0.16	3.77 ± 0.11	69.09 ± 0.07
PU-ZnO 0.25 wt %	35 ± 2	1.33 ± 0.26	4.26 ± 0.13	47.25 ± 0.15	1.32 ± 0.26	3.69 ± 0.11	63.68 ± 0.14	1.34 ± 0.25	3.50 ± 0.10	67.98 ± 0.13
PU-ZnO 0.50 wt %	36 ± 2	1.96 ± 0.28	3.81 ± 0.14	55.23 ± 0.17	1.48 ± 0.27	3.93 ± 0.12	69.31 ± 0.16	1.43 ± 0.24	3.81 ± 0.11	80.72 ± 0.17
PU-ZnO 0.75 wt %	38 ± 2	1.39 ± 0.28	3.79 ± 0.14	53.64 ± 0.17	1.50 ± 0.26	3.65 ± 0.11	58.07 ± 0.15	1.39 ± 0.25	3.55 ± 0.10	64.70 ± 0.15
PU-ZnO 1.0 wt %	38 ± 2	1.30 ± 0.28	3.30 ± 0.13	31.64 ± 0.15	1.28 ± 0.25	2.99 ± 0.11	42.72 ± 0.14	1.37 ± 0.24	3.00 ± 0.10	51.57 ± 0.14

**Table 5:** Kinetic diameter and condensability of gases.

Gas	Kinetic Diameter (Å)	Condensability (K)	Polarizability (Å)
CO_2_	3.30	195	1.9
CH_4_	3.80	149	2.6
N_2_	3.64	71	1.4

## Supplementary Materials

Supplementary File 1

## Abbreviations

PU	Polyurethane	
MMM	Mixed matrix membrane	
SS	Soft segments	
HS	Hard segments	
TGA	Thermogravimetric analysis	
AFM	Atomic-force microscopy	
SEM	Scanning electron microscope	
DMF	*N*,*N*-dimethylformamide	
*P*	Permeability	barrers
*Q*	Flux	cm^3^/s
*A*	Module area	cm^2^
*p*	Pressure	bar
t	Thickness	µm
*T*	Temperature	°C
(HBI)	Hydrogen Bonding Index	
