# Supplementary material for: Effects of ZnO Nanoparticle on the Gas Separation Performance of Polyurethane Mixed Matrix Membrane
Source: Membranes (Basel). 2017 Aug 11;7(3):43. doi: 10.3390/membranes7030043 (PMC5618128; doi:10.3390/membranes7030043)
Supplement: Supplementary File 1 [file membranes-07-00043-s001.pdf]

EDITORIAL CERTIFICATE LETTER

This document is to certify that the manuscript listed below was edited for proper English language, grammar, punctuation, spelling, and overall style by one of the highly qualified subject-expert native English speaking editors at **NativeEnglishEdit.com**

The substantive content of the article mentioned below remains the full responsibility of the author/authors:

TITLE OF ARTICLE:
EFFECTS OF ZNO NANOPARTICLE ON THE GAS SEPARATION PERFORMANCE OF POLYURETHANE MIXED MATRIX MEMBRANE

AUTHOR(S):
BANAFSHEH SOLTANI, MORTEZA ASGHARI

REFER CODE:
EE-1396-3334297-SOLTANI BANAFSHE 25103-1

Documents receiving this certification should be English-ready for publication; however, the author has the ability to accept or reject our suggestions and changes.
This certificate may be verified at:

Native English Edit
www.birminghamresearchpark.co.uk/tenants/native-english-edit
www.NativeEnglishEdit.com
Birmingham Research Park
Edgbaston
Birmingham B15 2SQ
United Kingdom
